# What Are the Key Elements for Achieving Well-Being Within the Framework of Healthy Aging?

**DOI:** 10.3390/healthcare14203506

**Published:** 2026-10-19

**Authors:** Víctor Manuel Mendoza-Núñez

**Affiliations:** Research Unit on Gerontology, FES Zaragoza, National Autonomous University of Mexico, Mexico City 09230, Mexico; mendovic@unam.mx

## 1. Introduction

Healthy aging (HA) is a priority objective, goal, and strategy for achieving maximum well-being and quality of life for older adults within the framework of community gerontology programs [1]. Prior to 2015, some studies inadequately treated terms such as the absence of non-communicable diseases (NCDs), active aging, successful aging, positive aging, or healthy lifestyles as synonyms for HA [2,3,4,5,6]. This led to confusion and made it impossible to determine its prevalence or propose specific strategies to achieve it, given the concept’s great heterogeneity.

In 2015, the World Health Organization (WHO) aptly and precisely established the construct and operational variables of HA in its “World Report on Ageing and Health” [7]. In this context, HA was defined as “the process of developing and maintaining the functional ability (FA) that enables wellbeing in older age” [7]. Furthermore, FA encompasses health-related attributes regarding activities of daily living (basic, instrumental, and advanced)—combined with a supportive environment—that enable a person to be and do what is important to them. Functional ability is determined by intrinsic capacity and the environment; the former comprises genetic inheritance, health status related to aging, personal characteristics, and lifestyle, and is identified through the combination of all a person’s physical and mental capabilities, while the environment includes housing characteristics, family dynamics, community participation, social programs, and public policies [1,7]. In this regard, the primary outcome of healthy aging is well-being and quality of life achieved through FA.

Within this framework, a Special Issue titled “Research on Health-Related Quality of Life and Well-being in Older Adults” was published, aiming to contribute to the study of factors and conditions that can positively influence the well-being or quality of life of older adults—within the framework of healthy aging—across various situations or environments. The eight articles published in this Special Issue address diverse topics related to HA, placing explicit or implicit emphasis on well-being and quality of life.

We invite all colleagues interested in the study of well-being, quality of life, and HA to comprehensively review the contents of this Special Issue; the topics presented offer insight into various facets and contexts of this field of study, as well as potential avenues for future research.

## 2. Development of the Field of Knowledge

As previously noted, it was not until 2015 that the WHO defined the construct of healthy aging, emphasizing the importance of maintaining and prolonging functional ability as a key element for achieving well-being [2]. Furthermore, in 2017, the WHO proposed the Integrated Care for Older People (ICOPE) model as an operational strategy for implementing programs for healthy aging, emphasizing the importance of Person-Centered Care (PCC). In this regard, ICOPE establishes guidelines for detecting priority conditions associated with declining intrinsic capacity and unsuitable environments, and for devising strategies and actions for HA across various contexts. This approach is PCC, taking into account the health, social care, and well-being needs of the individual, their family, and their caregiver (if applicable), rather than being limited to the management of NCDs or isolated symptoms [8]. For this reason, ICOPE focuses on the components of FA (intrinsic capacity and environment) with the following aims: (i) meeting basic needs, (ii) maintaining and improving mobility, (iii) creating and strengthening social support networks, (iv) learning new things for personal well-being, and (v) contributing to society. All these actions are aimed at achieving optimal health, well-being, and quality of life in old age [8].

Subsequently, in 2020, the WHO established the Decade of Healthy Ageing (2021–2030) strategy, identifying four areas of action to collectively foster healthy aging: (i) “changing how we think, feel, and act towards age and ageing,” with the aim of countering ageism and its negative impact on health and well-being; (ii) “ensuring that communities foster the abilities of older people,” to enhance their capabilities through a community gerontology approach; (iii) “delivering person-centred integrated care and primary health services that are responsive to older people,” aiming to optimize community resources by considering individualized care, peer support networks, self-care, and self-management; and (iv) “providing access to long-term care for older people who need it,” to improve the quality of care, well-being, and quality of life for older adults with functional limitations [9].

**Figure 1:**
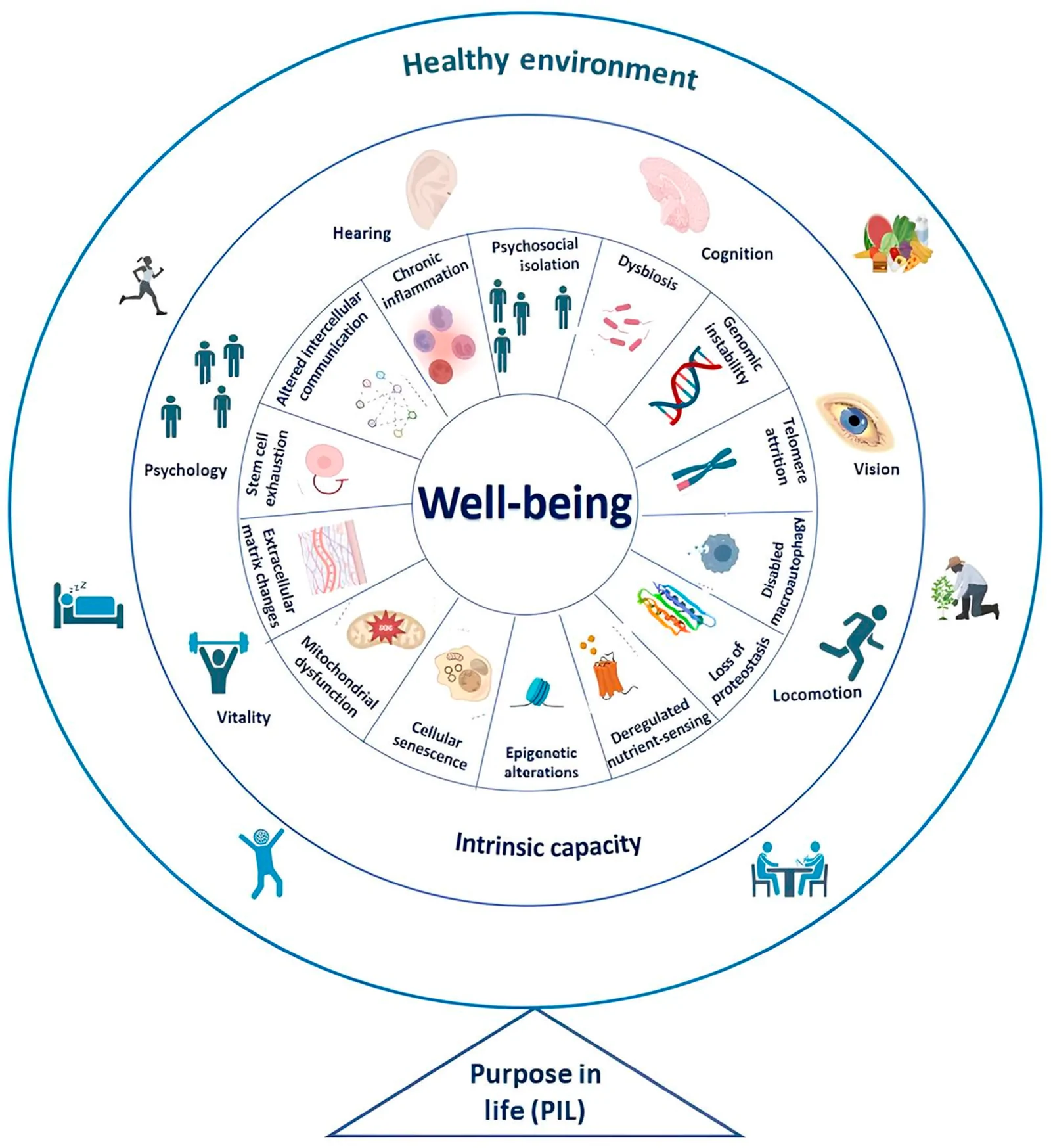
PIL and the environment as key elements of healthy aging. The scheme shows how the PIL combined with a healthy environment have a direct effect on the domains of the intrinsic capacity of the ICOPE, which influences the hallmarks of aging in a bidirectional manner and everything is linked to well-being.

In this context, we consider “purpose in life” (PIL) to be a key element and driver of healthy aging; when combined with a healthy environment, conjuntamente positively affects the ICOPE domains of intrinsic capacity. This leads to beneficial physiological, biochemical, and molecular changes regarding the hallmarks of aging, ultimately resulting in the attainment and maintenance of well-being (Figure 1). It is important to clarify that the proposed framework includes key elements for achieving well-being—a model that should be interpreted dynamically rather than through a hierarchical, unidirectional lens—since, although PIL serves as the foundation or initial link, we must bear in mind that PIL itself may also depend on the other components of our proposal.

### 2.1. Purpose in Life (PIL)

PIL can be defined as a person’s life objectives and goals, accompanied by reasoned actions or strategies to achieve them; it constitutes a key element of subjective well-being. In this regard, AshaRani et al. (2022)—in a systematic review of the conceptualization, measures, and determinants of PIL that analyzed 44 studies—identified six different approaches or meanings: (i) spirituality and religiosity, (ii) health and well-being, (iii) social relationships, (iv) meaningful objectives and goals, (v) importance to others, and (vi) inner strength [10]; however, it must be noted that PIL is individualized, contextual, and temporal, meaning that a combination of these meanings—with an emphasis on one or more of them—may be observed. Regarding the relationship between PIL and health status, better health has been observed in older adults who score higher on PIL measurement scales [11,12].

On the other hand, the Japanese concept of “Ikigai”—defined as “a reason for being” or “what makes life worth living”—has been proposed; while it shares many characteristics with FIL, “Ikigai” encompasses both eudaimonic and hedonic well-being, whereas FIL emphasizes the eudaimonic aspect above all [13]. In this regard, it has been reported that older adults with “Ikigai” exhibit better health status and functional ability, as well as fewer depressive symptoms and a lower risk of developing dementia [14].

### 2.2. Healthy Environment

**Figure 2:**
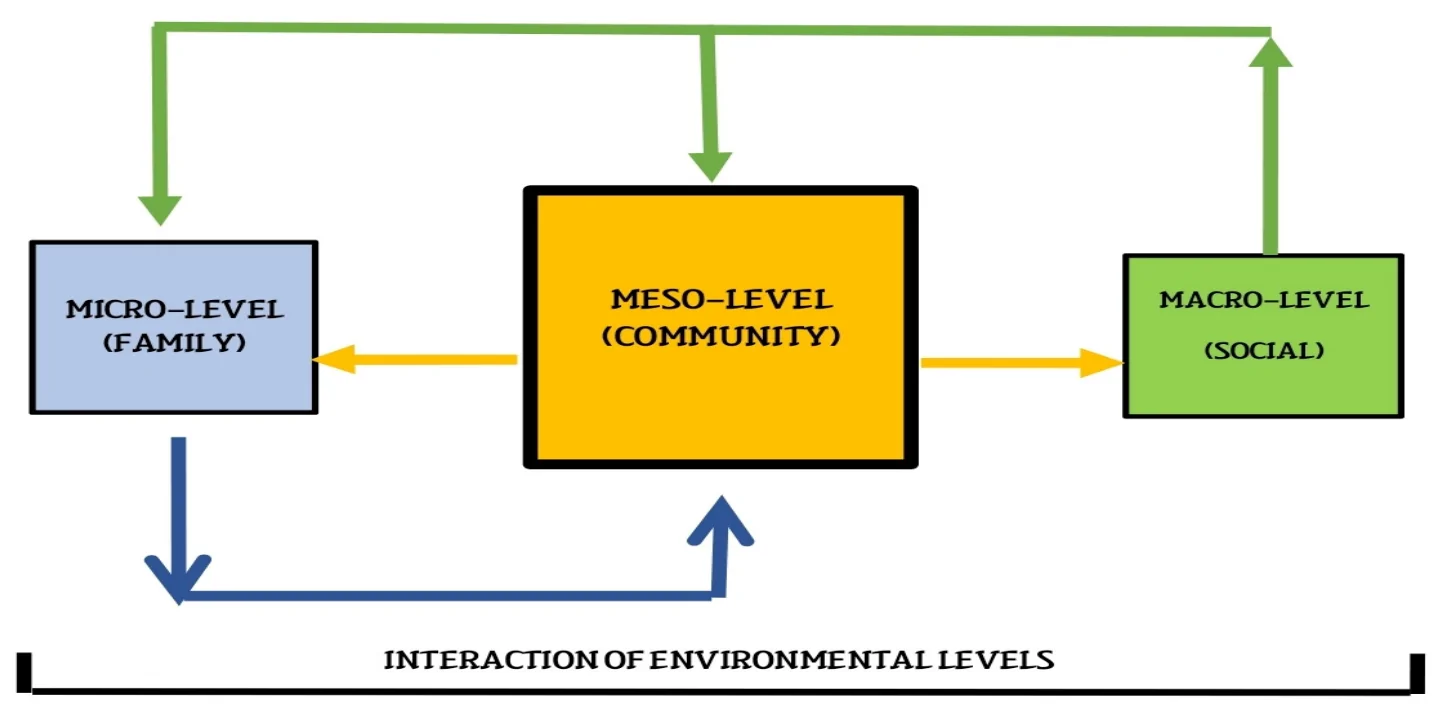
Levels of a healthy environment. The micro-level includes the characteristics of the home, the family, and their daily environment. The meso-level includes the community, specific programs for older adults, spaces for leisure and recreation, generative programs (social participation), and health services. The macro-level includes public policies, negative social representations of aging and old age (ageism), and recognition of the human and social capital of older adults for their own development. The colors and arrows show the influence of the different environmental levels on one another.

Another determinant of well-being is a healthy environment, which encompasses three levels: (i) the micro-level, housing characteristics and family interaction; (ii) the meso-level, community activities and programs for older adults; and (iii) the macro-level, social and health services linked to public policies (Figure 2) [15].

As previously noted, a healthy environment, combined with intrinsic capacity, determines functional capacity; and the ultimate outcome is well-being. In this regard, it must be considered that environments are not neutral in their relationship with people; indeed, the same environment can affect each individual in very different ways.

In 2007, the WHO proposed a “healthy city or community” (HC) model for older adults, describing it as an environment where services and physical structures are adapted to be more inclusive and to meet the needs of the older adult population. The HC model aims to foster HA by optimizing resources to improve health and functional ability, safety, and the active inclusion of older adults in the community. In this regard, the following distinctive characteristics of an HC were established: (i) providing older adults with more opportunities to participate in daily life, age healthily, and live without fear of discrimination or poverty; (ii) offering older adults the opportunity to grow and develop as individuals while contributing to their community; (iii) enabling older adults to age with dignity and autonomy; and (iv) fostering healthy aging through policies, services, environments, and structures [16].

Another concept related to healthy environments is that of “Blue Zones” (BZ)—geographic regions sharing similar lifestyles and environments where exceptional longevity (high percentage of centenarians) has been observed. Five communities in different countries are recognized as BZ: (i) Sardinia (Italy); (ii) Okinawa (Japan); (iii) Ikaria (Greece); (iv) Nicoya (Costa Rica); and (v) Loma Linda (USA) [17]. In this context, Buettner proposed nine distinctive lifestyle characteristics of BZ residents, which he termed the “Power 9^®^”: (i) vigorous physical activity; (ii) a sense of purpose in life (“ikigai”); (iii) reduced psychological stress; (iv) moderate food intake (stopping before feeling full, at 80% capacity, “Hara hachi bu”); (v) a diet rich in fruits, vegetables, and legumes; (vi) moderate alcohol consumption (1–2 glasses of wine per day); (vii) spirituality (specifically, belonging to a religious group); (viii) building and maintaining family ties (parents, siblings, children, grandchildren, and others); and (ix) healthy mutual-support groups (“moais”) [18].

Furthermore, our research group has developed a “gerontological nuclei” model—considered a community environment conducive to healthy aging—that recognizes the human and social capital represented by older adults in order to enhance their functional ability and human development. To this end, older adults undergo formal training as “healthy aging health promoters” to actively encourage self-care and mutual-help “peer-to-peer”, fostering the adoption and maintenance of healthy lifestyles that preserve and extend functional ability, thereby achieving optimal well-being and quality of life [19].

### 2.3. ICOPE Domains

The ICOPE domains for assessing intrinsic capacity established by the WHO are as follows: (i) locomotor capacity (mobility); (ii) vitality (nutrition); (iii) sensory capacity (visual and auditory); (iv) cognitive capacity; and (v) depressive symptoms [8]. However, our research group has proposed adding five further domains: (i) gustatory and olfactory capacity; (ii) oral health; (iii) sleep; (iv) social participation; and (v) purpose in life [1]. It is important to note that all domains are interdependent; therefore, it is necessary to propose multidimensional community-based interventions.

### 2.4. Biological and Psychosocial Integration of the Hallmarks of Aging

In 2013, López-Otín et al. proposed nine hallmarks of aging: (i) genomic instability; (ii) telomere attrition; (iii) epigenetic alterations; (iv) loss of proteostasis; (v) altered metabolic regulation; (vi) mitochondrial dysfunction; (vii) cellular senescence; (viii) stem cell exhaustion; and (ix) altered intercellular communication [20]. Subsequently, in 2023, these researchers added three more: (i) macroautophagy; (ii) dysbiosis; and (iii) chronic inflammation [21]; in 2025, they included two additional ones: (i) extracellular matrix changes; and (ii) psychosocial isolation [22]. Consequently, fourteen hallmarks of aging are currently recognized, and the importance of psychosocial aspects as a key element embedded within biological and molecular mechanisms has been highlighted; thus, the current approach to studying the hallmarks of aging acknowledges the mutual influence between biological-molecular mechanisms and psychosocial processes [23,24]. This comprehensive view of the hallmarks of aging presents a challenge for conducting multidisciplinary translational research.

### 2.5. Well-Being and Quality of Life

Regarding well-being, two types or approaches have been distinguished: the hedonic and the eudaimonic. The hedonic approach—or subjective well-being—is interpreted as the attainment of happiness through maximizing pleasure and minimizing unpleasant experiences; it is perceived as short-term and intermittent, encompassing life satisfaction, positive affect, and a reduction in negative affect. The eudaimonic approach—or psychological well-being—posits that well-being stems from human potential and development, enabling individuals to realize projects they find fulfilling; consequently, it is perceived over a longer timeframe, as it encompasses the plan, the process, and the outcome, with self-acceptance, personal growth, autonomy, and a sense of purpose in life serving as key determinants [25]. Both types of well-being are closely interrelated and complementary; therefore, community interventions aimed at healthy aging seek to foster both forms of well-being.

On the other hand, the concept of quality of life is highly heterogeneous, as it represents a personal, contextual, and temporal perception determined by external factors—such as economic situation, education, place of residence, employment, and family and social relationships—alongside individual aspects such as physical and mental health status, sex, age, functional capacity, spirituality, religiosity, and life expectations [26]. In 2002, within the framework of active aging, the WHO defined quality of life as “an individual’s perception of his or her position in life in the context of the culture and value system where they live, and in relation to their goals, expectations, standards and concerns. It is a broad ranging concept, incorporating in a complex way a person’s physical health, psychological state, level of independence, social relationships, personal beliefs and relationship to salient features in the environment” [27].

Well-being is the primary outcome of healthy aging—an outcome resulting from functional ability, which is determined by intrinsic capacity and the environment. In this regard, the hallmarks of aging have been linked to well-being in older adults [22,28]; consequently, we must view healthy aging “from the cell to the community” to promote a holistic, translational approach.

## 3. Contributions of the Special Issue

The theoretical framework presented in this editorial offers a broad, inclusive perspective on Healthy Aging, highlighting the importance of a holistic approach that considers biological and psychosocial factors across various contexts. It emphasizes well-being as the primary outcome—a factor either explicitly measured or implicitly inferred in the eight articles featured in this Special Issue, which present findings on healthy lifestyle factors and health status amidst the diverse situations and experiences of older adults across different settings and countries.

A summary of each of the eight contributions to this Special Issue is presented below.

Sleep quality is one of the ICOPE domains proposed by our research group, given that adequate sleep has been shown to increase the likelihood of healthy aging [29]. In this context, Chen et al. (Contribution 1) conducted an analytical cross-sectional study using a non-probability sample of 850 individuals aged 50 and older (243 men and 607 women) to determine the relationship between sleep quality and health status. They measured sleep quality using the Pittsburgh Sleep Quality Index and considered ≥7 h to be the optimal sleep duration. They also assessed self-reported health status, categorizing it as good, fair, or poor. The authors found greater dissatisfaction with health status among participants with poor sleep quality, a finding linked to the lower number of sleep hours reported per day.

Frailty is a construct used to clinically classify older adults with diminished intrinsic capacity—particularly vitality—resulting from reduced physiological reserve and homeostatic response; this manifests as a significant decline in functional ability and is associated with an increased risk of falls, hospitalization, and the need for personalized long-term care [30]. In this context, Pérez-Saiz et al. (Contribution 2) assessed the holistic health and well-being of 240 frail older adults from seven primary care centers in Valencia, Spain. Their descriptive cross-sectional study was conducted as part of the “ValueCare” project, which aims to deliver personalized, integrated health and social care and achieve better outcomes for older people. The authors note that their findings differed from those of other studies but were relevant for adapting the “ValueCare” model to the Valencian community, taking into account the specific characteristics of the city’s older population.

Loneliness and social isolation have been identified as risk factors for physical and mental health in old age [31]. In this context, Hung et al. (Contribution 3) conducted a qualitative study involving 19 older adults (5 men and 14 women) in Hong Kong to explore the lived experiences of loneliness using interpretative phenomenological analysis. Addressing the question “Should I Stay at Home Alone? Lived Experiences of Loneliness,” the authors note that participants expressed a desire to avoid being alone or socially isolated; however, physical, social, familial, and emotional factors often prevented this desire from being realized. Consequently, the authors suggest the need for public policies that encourage government and non-government social and health institutions to implement comprehensive programs—involving social support networks such as family and the community—to promote healthy aging among older adults living in loneliness.

Self-care based on an active aging approach—utilizing a “peer-to-peer” mutual-help strategy—has been proposed as a means to promote healthy aging through social support networks [19]. In this context, Zhu & Li (Contribution 4) conducted an exploratory study to identify social support characteristics associated with online health information seeking (OHIS) among older adults in China, specifically comparing “patient-to-doctor” and “patient-to-peer” platforms. The authors found that “patient-to-peer” platforms provide stronger emotional support and foster more meaningful online relationships and emotional empathy than “patient-to-doctor” platforms. Conversely, “patient-to-doctor” platforms are more focused on treatment outcomes.

A family environment and person-centered care are key factors in promoting and achieving healthy aging at home [1], hence the importance of recognizing the needs perceived by both the care recipient and the caregivers. A cross-sectional study by Szewczyczak et al. (Contribution 5) presents findings regarding the perceived needs of 193 older adults (≥75 years) receiving home-based care in Poland, comparing these with the needs identified by a nurse-researcher (the study’s lead author) for the same group of patients and those reported by 97 caregivers. Notably, the most significant finding was that researchers and caregivers identified more met and unmet needs than the elderly individuals themselves.

Intergenerational interventions have been reported to have a positive effect on certain psychosocial variables linked to well-being in old age [32]. In this regard, a study by Wang et al. (Contribution 6)—conducted with 2970 older adults aged 60 and over from the China Health and Retirement Longitudinal Study (CHARLS)—not only explored the relationship between intergenerational care and life satisfaction but also analyzed the chain-mediating effect of emotional support from children and depression on this relationship, thereby confirming the association.

Physical exercise is a key component of a healthy lifestyle throughout the life course; in old age, it has a positive, cross-cutting effect on the domains of intrinsic capacity defined by the ICOPE framework [8]. Consequently, various studies have examined the barriers to and motivations for initiating and maintaining long-term adherence to physical exercise, identifying environmental, psychosocial, and economic factors as primary influences [33]. Hwang et al. (Contribution 7) conducted a cross-sectional study involving 1000 South Korean adults ≥ 50 years to “investigate how perceived exercise barriers, financial burden, constraint negotiation mechanisms, and social support influence exercise adherence … by integrating behavioral economics, constraint negotiation theory, and social cognitive theory.” The authors observed that perceived exercise barriers and financial burden were negatively associated with exercise adherence, whereas constraint negotiation mechanisms and perceived social support were positively associated. Mediation analyses revealed that constraint negotiation strategies partially mediated the relationships between perceived barriers and adherence, as well as between financial burden and adherence. Moderation analysis confirmed that social support buffered the negative effects of financial and psychological barriers.

Tai Chi (TC) training is recommended as a form of physical exercise to improve balance and prevent falls in older adults; however, its effect on muscle mass in terms of preventing the progression of sarcopenia remains controversial [34]. Consequently, Flores-Bello et al. (Contribution 8) conducted a quasi-experimental study using a convenience sample of 68 older Mexican women to determine the effects of TC training—compared to strength training (ST)—on skeletal muscle mass, physical performance, cognitive function, and psychological well-being. The authors found that TC has a greater effect than ST on skeletal muscle mass, which is linked to handgrip strength and physical performance. Additionally, the percentage change in psychological well-being was statistically significant in both groups; however, cognitive function showed no changes.

## 4. Future Research Directions

Purpose in life, combined with a healthy environment, constitutes a key determinant and element for achieving well-being within the framework of healthy aging [11,12,13,14,15]. Therefore, it is advisable to include the assessment of these two variables and to propose intervention strategies to strengthen or improve them in studies on well-being and healthy aging.

On the other hand, person- and community-centered care is another variable that must be considered in studies [8]. In this regard, measuring and monitoring the effects of interventions while taking into account individuality and personal interests is fundamental to assessing well-being and quality of life.

Integrating the hallmarks of aging—including biological and molecular mechanisms—with psychosocial factors is one of the most recent proposals for extending healthspan and achieving optimal well-being in old age through translational research [22,23,24]. Therefore, we must build bridges between basic, clinical, and psychosocial researchers to conduct holistic, multidimensional research and gain a less fragmented understanding of healthy aging.

## 5. Conclusions

The study of healthy aging is broad and inclusive; regardless of health status or physical limitations, the construct posits that by improving intrinsic capacity (to the extent possible) within a suitable environment, functional capacity can be enhanced or optimized to achieve maximum well-being and quality of life.

Overall, this Special Issue presents various approaches to healthy aging research, emphasizing the assessment of well-being—whether implicitly or explicitly—as a primary or secondary outcome. Furthermore, the collection of articles exemplifies potential research directions in this field, featuring topics that could be replicated or adapted in other contexts.
